# Mixed-Ligand Engineering to Enhance Luminescence of Mn^2+^-Based Metal Halides for Wide Color Gamut Display

**DOI:** 10.3390/ma17184459

**Published:** 2024-09-11

**Authors:** Zhi Wu, Huidong Tang, Tianhao Dai, Yuxi Long, Dan Luo, Pengcheng Jiang, Xin Xiong, Yanqiao Xu, Xiaojun Zhang, Qing Hu

**Affiliations:** 1 School of Material Science and Engineering, Hunan Institute of Technology, Hengyang 421002, China; wuzhi0549@163.com (Z.W.); 19350382164@163.com (T.D.); 15183673851@163.com (Y.L.); 17383840952@163.com (D.L.); wustjpc@163.com (P.J.); xwustx@163.com (X.X.); 2 National Engineering Research Center for Domestic & Building Ceramics, Jingdezhen Ceramic University, Jingdezhen 333001, China; xuyanqiao@jcu.edu.cn (Y.X.); zhangxiaojun@jci.edu.cn (X.Z.); huqing@jci.edu.cn (Q.H.); 3 School of Material Science and Engineering, Jingdezhen Ceramic University, Jingdezhen 333001, China

**Keywords:** Mn^2+^-based metal halide, phosphors, PL properties, mixed-ligand strategy, stability

## Abstract

Lead-free Mn^2+^-based metal halide materials are now being considered as clean candidates for backlight displays and lights due to the d–d transition, diverse components, and environmental friendliness. Therefore, efficient and stable Mn^2+^-based metal halide phosphors are in great demand for practical applications. In this work, adopting the mixed-ligand strategy, a series of [(CH_3_)_4_N]_2−*x*_[(C_2_H_5_)_4_N]*_x_*MnCl_4_ phosphors were synthesized by mechanochemical process. With the increase molar ratio of (CH_3_)_4_N/(C_2_H_5_)_4_N, the phase of phosphors is transformed from orthorhombic to tetragonal. Compared to [(CH_3_)_4_N]_2_MnCl_4_ and [(C_2_H_5_)_4_N]_2_MnCl_4_ phosphors, the mixed-ligand strategy significantly boosts the green emission intensity of Mn^2+^-based metal halide phosphors. The obtained [(CH_3_)_4_N][(C_2_H_5_)_4_N]MnCl_4_ phosphors exhibit a high photoluminescence quantum yield (PLQY) of 83.78% under 450 nm excitation, which is attributed to the modulation of the adjacent [MnCl_4_]_2_- distance by using the different chain length of organic cations, effectively suppressing non-radiative recombination. Additionally, the [(CH_3_)_4_N][(C_2_H_5_)_4_N]MnCl_4_ phosphors exhibit a green emission at 516 nm, narrow full width at half-maximum (FWHM) of 45.53 nm, and good thermal stability. The constructed white light-emitting diode (WLED) device exhibits a wide color gamut of 108.3% National Television System Committee, demonstrating the suitability of the [(CH_3_)_4_N][(C_2_H_5_)_4_N]MnCl_4_ phosphors as a green emitter for WLED displays and lightings. This work provides a new way to modulate the PL performance of manganese-based metal halides for application in the optoelectronic field.

## 1. Introduction

Recently, phosphor-converted white light-emitting diodes (WLEDs) have been recognized as an outstanding backlight unit in liquid crystal displays (LCDs), addressing the demand for high color purity and brightness, low power consumption, and long operation lifetime [1,2,3]. Green phosphors with a narrow full width at half maximum (FWHM) are one of the important components for the wide color gamut of LCD displays [4,5]. Commercially, *β*-sialon: Eu^2+^ phosphor, as a green emitter, has been widely applied in backlight displays, exhibiting an emission wavelength of 540 nm and a FWHM of 55 nm, resulting in a color gamut of less than 90% of the National Television Systems Committee (NTSC) in the CIE 1931 color space [6]. Subsequently, Eu^2+^-activated phosphors (such as RbLi(Li_3_SiO_4_)_2_:Eu^2+^ [7], Na_3_K_5_(Li_3_SiO_4_)_8_:Eu^2+^ [8], (Sr, Ba)_2_SiO_4_:Eu^2+^ [9], etc.) and Mn^2+^-doped phosphors (including Sr_2_MgAl_22_O_36_:Mn^2+^ [10], NaGa_11_O_17_:Mn^2+^ [11], Zn_2_GeO_4_:Mn^2+^ [12], etc.) have been synthesized. Unfortunately, the poor stability and harsh synthesis conditions prevent them from being commercially viable on a large scale. In addition, green-emitting APbBr_3_ (A = Cs, CH_3_NH_3_^+^, and CH_2_(NH_2_)_2_^+^) metal halide perovskite nanocrystals with a high photoluminescence quantum yield (PLQY = ~100%) and a narrow FWHM (~20 nm) have piqued the attention of researchers [13,14]. Nevertheless, the poor light and thermal stability of APbBr_3_ nanocrystals, coupled with the inherent toxicity of lead, hinder their practical applications [15,16]. Therefore, the exploration of new green phosphors with efficient emission, narrow emission line-width, good stability, environmental friendliness, and low manufacturing consumption is crucial for achieving large-scale backlight display applications.

Recently, manganese-based metal halides as green emitters have made impressive progress due to their extremely sensitive d–d transition for crystal field and environmental friendliness [17,18]. For example, Fu et al. [19] prepared o-C_44_H_38_P_2_MnBr_4_ (o-C_44_H_38_P_2_ = 1,2-phenylenebis(methylene))bis(triphenylphosphonium) single crystals with [MnBr_4_]^2−^ tetrahedra, which exhibit a strong green emission at 517 nm with the FWHM of 43 nm and a PLQY of 95.93% under 450 nm excitation. Zhang et al. [20] reported C_16_H_38_N_2_MnBr_4_ (C_16_H_38_N_2_ = decamethonium) single crystals prepared by the slow evaporation method, which show a green emission at 534 nm with a PLQY of 76% under an excitation wavelength of 460 nm. They also fabricated a WLED device with a wide color gamut of 108% of NTSC. Manganese-based metal halides exhibit a narrow FWHM and high PLQY, making them excellent candidates for application in LCD backlight displays [21,22]. However, the synthesis of manganese-based metal halide single crystals typically involves the cooling crystallization method or solution evaporation method, both of which require the use of significant amounts of acid waste liquid or organic liquid and involve harsh synthesis conditions [23,24,25]. To address this problem, mechanochemical and facile solution-processed methods have been developed to prepare manganese-based metal halide phosphors [26,27], offering the advantages of being environmentally friendly, having moderate reaction conditions, and allowing for large-scale synthesis. For instance, Peng et al. [28] prepared C_8_H_20_N_2_MnBr_4_ (C_4_H_10_N = pyrrolidine) powders via mechanochemical synthesis, which exhibit a green emission at 520 nm and a PLQY of 19%. Ma et al. [29] used the mechanochemical method to prepare [EMMIM]_2_MnCl_4_ powders by using 1-ethyl-2,3-dimethylimidazolium (EMMIM) bromide and MnBr_2_·4H_2_O, which exhibit an emission at 511 nm and a PLQY of 79.5%. Unfortunately, the emission properties of manganese-based metal halide phosphors show a lower efficiency than that of single crystals due to the presence of more defect states and the inherent d–d spin transitions of Mn^2+^. To modulate the emission properties of phosphors, the adjustable interactions between adjacent Mn^2+^ ions strategy is proposed by using different organic cations [30]. For instance, Mao et al. [30] used small cations (dimethylammonium, DDA) and large cations (tetraphenylphosphonium, PPh_4_) to prepare (DMA)_2_MnBr_4_ and (PPh_4_)_2_MnBr_4_ single crystals with the shortest Mn–Mn distances of 6.22 Å and 10.45 Å, achieving the PLQY of 7.8% and 98%, respectively, which revealed that a large and bulky organic cation was in favor of achieving efficient emission. Pan et al. [31] prepared a series of manganese-based metal halide phosphors by rationally managing the organic cations, resulting in an increase in the Mn–Mn distances from 7.90 Å to 9.492 Å, and enhancing the PLQY from 7.98% to 81.11%. Hence, the effective radius of the organic cations plays a key role in modulating the Mn–Mn distances. Based on the above reports, the luminescence properties of manganese-based halide phosphors can be further boosted by developing a desirable organic cation ligand strategy, making them meet the desired standards for application in backlight displays.

In this study, the mixed-ligand strategy based on the different chain length of organic cations was developed to improve the emission intensity of metal halide phosphors prepared by a facile mechanochemical method. The effect of n[(CH_3_)_4_N]/n[(C_2_H_5_)_4_N] on the structure and PL properties was systematically investigated. It was found that the PLQY improved from 13.48% of [(CH_3_)_4_N]_2_MnCl_4_ phosphors to 83.78% of [(CH_3_)_4_N][(C_2_H_5_)_4_N]MnCl_4_ phosphors. The fluorescence enhancement mechanism and stability of [(CH_3_)_4_N][(C_2_H_5_)_4_N]MnCl_4_ phosphors were discussed. Subsequently, [(CH_3_)_4_N][(C_2_H_5_)_4_N]MnCl_4_ phosphors and commercialized K_2_SiF_6_ phosphors were mixed and coated on a blue InGaN chip to fabricate a WLED device, which exhibited a high luminous efficacy of 66.34 lm/W and a wide color gamut of 108.3% NTSC. This indicates that [(CH_3_)_4_N][(C_2_H_5_)_4_N]MnCl_4_ phosphors have great potential as a green emitter for displays and lighting fields.

## 2. Experiment and Characterization

### 2.1. Materials

Tetramethylammonium chloride ((CH_3_)_4_NCl, 98%), manganese (II) chloride tetrahydrate (MnCl_2_·4H_2_O, 99%), ethanol (C_2_H_5_OH, 99.5%), and tetraethylammonium chloride ((C_2_H_5_)_4_NCl, 98%) were acquired from Shanghai Aladdin Biochemical Technology Co., Ltd., Shanghai, China. No further purification was carried out on any of the chemical reagents.

### 2.2. Synthesis of [(CH_3_)_4_N]_2−x_[(C_2_H_5_)_4_N]_x_MnCl_4_ Phosphors

The synthesis procedure for [(CH_3_)_4_N]_2−*x*_[(C_2_H_5_)_4_N]*_x_*MnCl_4_ phosphors followed a facile mechanochemistry process. To prepare [(CH_3_)_4_N]_2_MnCl_4_ phosphors, 2 mmol (CH_3_)_4_NCl and 1 mmol MnCl_2_·4H_2_O were placed into an agate mortar and manually ground for 30 min to promote complete chemical reactions. The reaction mixture was then washed with 5 mL of ethanol and the product was collected via centrifugation at 5000 rpm for 5 min. The mixture was washed three times and then dried at 70 °C for 2 h. [(CH_3_)_4_N][(C_2_H_5_)_4_N]MnCl_4_ and [(C_2_H_5_)_4_N]_2_MnCl_4_ phosphors were synthesized using the same preparation scheme by adjusting the (CH_3_)_4_N]/(C_2_H_5_)_4_N precursor molar ratio (*x*). To prepare [(CH_3_)_4_N][(C_2_H_5_)_4_N] MnCl_4_ phosphors, 1 mmol (CH_3_)_4_NCl, 1 mmol (C_2_H_5_)_4_NCl, and 1 mmol MnCl_2_·4H_2_O were employed. For the preparation of [(C_2_H_5_)_4_N]_2_MnCl_4_ phosphors, 2 mmol (C_2_H_5_)_4_NCl and 1 mmol MnCl_2_·4H_2_O were used.

### 2.3. Preparation of WLED Device

A WLED device was fabricated by combining the as-prepared [(CH_3_)_4_N][(C_2_H_5_)_4_N]MnCl_4_ green phosphors, commercial K_2_SiF_6_:Mn^4+^ red phosphors (Nanjing Jinhui Fluorescent Material Co., Ltd., Nanjing, China), and a blue InGaN chip (~450 nm). The green and red phosphors were mixed with UV-curable silicone, coated onto the InGaN chip, and solidified for 10 min to produce the WLED device.

### 2.4. Characterization

Powder X-ray diffraction (XRD) patterns were recorded on a Bruker D8 Advance diffractometer (Bruker, Billerica, MA, USA) with a Cu-Kα ceramic X-ray tube. Scanning electron microscopy (SEM) images and X-ray spectroscopy (EDS) were obtained using a ZEISS Gemini SEM 300 (Carl Zeiss AG, Oberkochen, Germany). PL properties were collected using an Edinburgh FLS1000 fluorescence spectrometer (Edinburgh, Scotland, UK), which included PL spectra, PL excitation (PLE) spectra, PLQY, PL decay curves, and temperature-dependent PL emission spectra. UV–Vis absorption spectra were measured using a Shimadzu UV-3600 (Shimadzu, Kyoto, Japan) with BaSO_4_ as a reference. The LED device properties were acquired using an Everfine ATA100 spectroradiometer (Everfine Opto-Electronic Technology Co., Ltd., Hangzhou, China).

### 2.5. First Principles Calculations

Density functional theory (DFT) calculations were performed using CASTEP 6.0 [32,33]. The [(CH_3_)_4_N][(C_2_H_5_)_4_N]MnCl_4_ system was constructed with a 1 × 1 × 1 supercell containing 204 atoms. The crystal structure was optimized, and the bandgap and electronic structure were obtained using the generalized gradient approximation (GGA) with Perdew–Burke–Ernzerhof (PBE) functional. A k-mesh of 2 × 2 × 2 was used for Brillouin zone integration. The cutoff energy and self-consistent-field (SCF) tolerance were set to 450 eV and 10^−5^ eV, respectively. The maximum force on any relaxed atom was 0.02 eV Å^−1^. The weak interaction was modeled using the DFT + D3 method, which incorporates an empirical correction based on Grimme’s scheme. Spin polarization was employed to characterize the magnetic system.

## 3. Results and Discussion

### 3.1. The Structure and Morphology of [(CH_3_)_4_N]_2−x_[(C_2_H_5_)_4_N]_x_MnCl_4_ Phosphors

**Figure 1:**
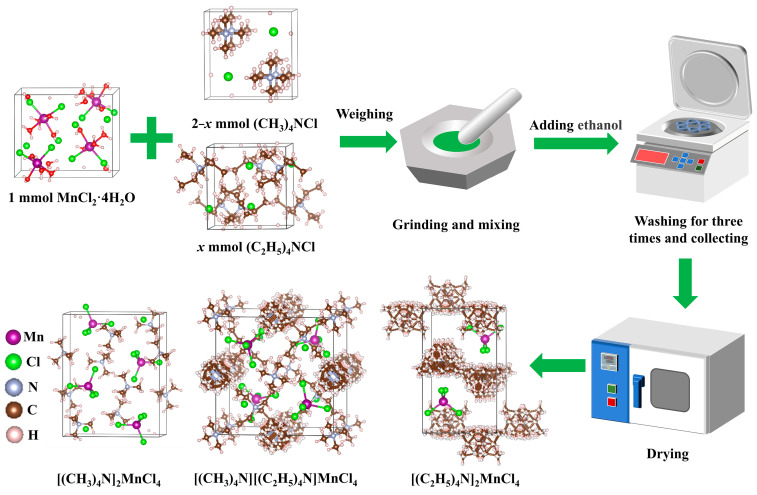
Scheme of [(CH_3_)_4_N]_2−*x*_[(C_2_H_5_)_4_N]*_x_*MnCl_4_ phosphors via facile mechanochemistry method and crystal structure.

The process of preparing [(CH_3_)_4_N]_2−*x*_[(C_2_H_5_)_4_N]*_x_*MnCl_4_ phosphors through a facile mechanochemistry method is illustrated in Figure 1. This method is widely used for the synthesis of metal halide perovskite materials such as Rb_2_ZrCl_6−*x*_Br*_x_* [34], Cs_3_Cu_2_I_5_ [35], CsPbX_3_ nanocrystals [36], etc. The [(CH_3_)_4_N]_2−*x*_[(C_2_H_5_)_4_N]*_x_*MnCl_4_ phosphors were directly obtained by milling a mixture of raw materials including (CH_3_)_4_NCl, (C_2_H_5_)_4_NCl, and MnCl_2_·4H_2_O, followed by washing and drying. The grinding process provides the optimal conditions for efficient mechanochemical reactions to occur, such as the high impact force for accelerating diffusion and shorting the diffusion time, as well as easy stoichiometry control [37]. The detailed synthesis process is depicted in the Section 2. The as-prepared [(CH_3_)_4_N]_2−*x*_[(C_2_H_5_)_4_N]*_x_*MnCl_4_ phosphors exhibit a 0D structure, in which one Mn atom is surrounded by four chlorine atoms to form the [MnCl_4_]^2−^ tetrahedron, while large (CH_3_)_4_N^+^ and (C_2_H_5_)_4_N^+^ organic cations occupy the interstices around the tetrahedron. The unique 0D structure gives it excellent optoelectronic properties.

**Figure 2:**
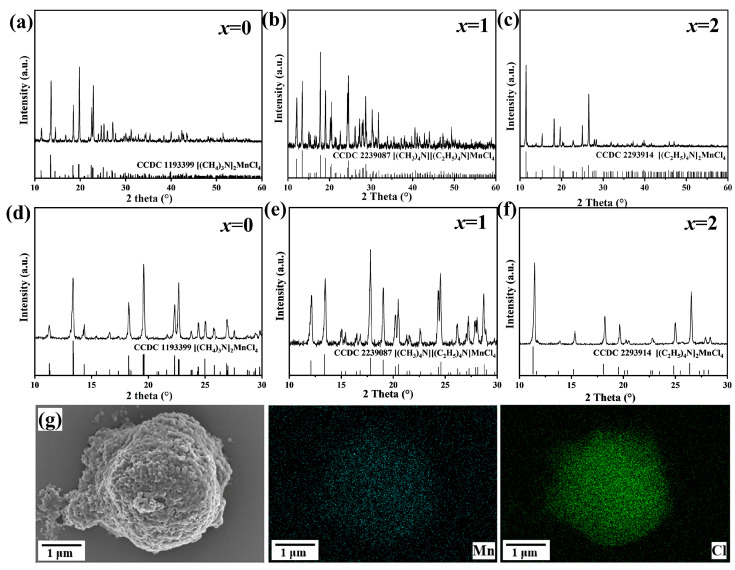
XRD patterns of [(CH_3_)_4_N]_2−*x*_[(C_2_H_5_)_4_N]*_x_*MnCl_4_ phosphors with different (CH_3_)_4_N/(C_2_H_5_)_4_N molar ratio (**a**–**f**), where (**d**–**f**) are the zoomed-in XRD patterns of (**a**–**c**) in the diffraction peak region of 10~30°, respectively. SEM image as well as EDS mapping (**g**) of [(CH_3_)_4_N][(C_2_H_5_)_4_N]MnCl_4_ phosphors (*x* = 1).

To explore the phase structure, XRD patterns of as-prepared [(CH_3_)_4_N]_2−*x*_[(C_2_H_5_)_4_N]*_x_*MnCl_4_ phosphors with a different n[(CH_3_)_4_N]/n[(C_2_H_5_)_4_N] precursor molar ratio were collected, as shown in Figure 2a–f. When *x* = 0, the XRD diffraction peaks match well with the standard pattern of [(CH_3_)_4_N]_2_MnCl_4_ (CCDC 1193399), demonstrating that [(CH_3_)_4_N]_2_MnCl_4_ belongs to the orthorhombic phase with *Pmcn* space group (Figure 2a). With an increase in the molar ratio to *x* = 1, the XRD diffraction peaks of the as-prepared sample can be well-indexed into the tetragonal [(CH_3_)_4_N][(C_2_H_5_)_4_N]MnCl_4_ structure (CCDC 2239087) with the *P*42_1_*m* space group (Figure 2b). When the (CH_3_)_4_N^+^ organic cations are completely replaced by (C_2_H_5_)_4_N^+^, the diffraction peaks obtained closely match the standard pattern of the tetragonal [(C_2_H_5_)_4_N]_2_MnCl_4_ structure (CCDC 2293914) with the *P*4_2_/*nmc* space group (Figure 2c). This change is because the effective radius of the (C_2_H_5_)_4_N^+^ organic cation (3.08 Å) is larger than that of the (CH_3_)_4_N^+^ organic cation (2.51 Å) [38,39]. And, as x increases, no additional diffraction peaks are observed (Figure 2d–f). The morphology and distribution of elements in the [(CH_3_)_4_N][(C_2_H_5_)_4_N]MnCl_4_ phosphors were analyzed using SEM and EDS mapping, as depicted in Figure 2d. It is observed that the Mn and Cl elements are evenly distributed within the particles. These results indicate the successful preparation of pure [(CH_3_)_4_N]_2−*x*_[(C_2_H_5_)_4_N]*_x_*MnCl_4_ phosphors through mechanochemistry.

### 3.2. PL Properties of [(CH_3_)_4_N]_2−x_[(C_2_H_5_)_4_N]_x_MnCl_4_ Phosphors

**Figure 3:**
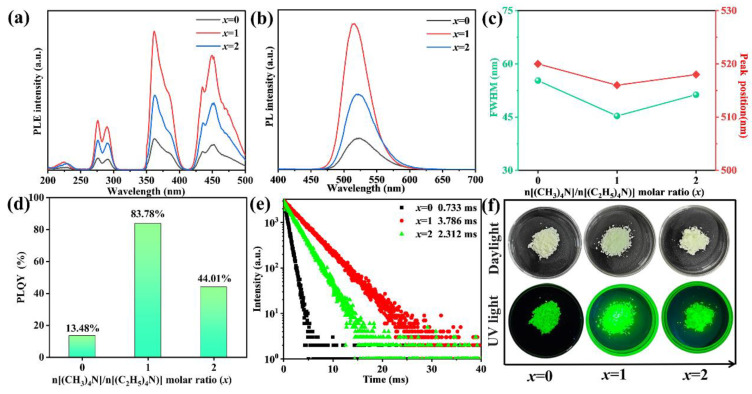
PLE spectra (**a**) and PL spectra (**b**) of [(CH_3_)_4_N]_2−*x*_[(C_2_H_5_)_4_N]*_x_*MnCl_4_ phosphors with different n[(CH_3_)_4_N]/n[(C_2_H_5_)_4_N], FHWM, and peak position vs. n[(CH_3_)_4_N]/n[(C_2_H_5_)_4_N] (**c**), PLQY (**d**), PL decay curves (**e**), and digital photographs (**f**) of [(CH_3_)_4_N]_2−*x*_[(C_2_H_5_)_4_N]*_x_*MnCl_4_ phosphors with different n[(CH_3_)_4_N]/n[(C_2_H_5_)_4_N].

To investigate the effect of n[(CH_3_)_4_N]/n[(C_2_H_5_)_4_N] substitution on the photoluminescent properties of [(CH_3_)_4_N]_2−*x*_[(C_2_H_5_)_4_N]*_x_*MnCl_4_ phosphors, the PLE spectra of [(CH_3_)_4_N]_2−*x*_[(C_2_H_5_)_4_N]*_x_*MnCl_4_ phosphors with different [(CH_3_)_4_N]/[(C_2_H_5_)_4_N] molar ratios (*x*) are depicted in Figure 3a. When the n[(CH_3_)_4_N]/n[(C_2_H_5_)_4_N] ratio is 0, four excitation bands of [(CH_3_)_4_N]_2_MnCl_4_ phosphors can be directly observed at 200~250 nm, 250~325 nm, 325~410 nm, and 410~500 nm under the emission of 520 nm. The excitation bands at 325~410 nm and 410~500 nm have strong excitation peaks, indicating that the prepared [(CH_3_)_4_N]_2_MnCl_4_ phosphors are a good match for commercial UV and blue LED chips. In the range of 325~410 nm, the bands around 361 nm and 384 nm are ascribed to the ^6^A_1_(S) → ^4^T_1_(P) and ^6^A_1_(S) → ^4^E(D) transitions of Mn^2+^, respectively. The bands at 410~500 nm have three excitation peaks around 435 nm, 452 nm, and 471 nm, which come from the ^6^A_1_(S) → [^4^A_1_, ^4^E(G)], ^6^A_1_(S) → ^4^T_2_(G), and ^6^A_1_(S) → ^4^T_1_(G) transitions of Mn^2+^ [21,22], respectively. With the increase in n[(CH_3_)_4_N]/n[(C_2_H_5_)_4_N], the excitation bands of the as-prepared [(CH_3_)_4_N][(C_2_H_5_)_4_N]MnCl_4_ and [(C_2_H_5_)_4_N]_2_MnCl_4_ phosphors show a nearly identical profile. The excitation peaks at 452 nm of the as-prepared [(CH_3_)_4_N][(C_2_H_5_)_4_N]MnCl_4_ and [(C_2_H_5_)_4_N]_2_MnCl_4_ phosphors have slightly shifted to 450 nm and 451 nm, respectively, due to the change in the crystal field for the Mn^2+^ environment with the modulation of the A-site ligands.

The PL spectra of [(CH_3_)_4_N]_2−*x*_[(C_2_H_5_)_4_N]*_x_*MnCl_4_ phosphors with a different n[(CH_3_)_4_N]/n[(C_2_H_5_)_4_N] are shown in Figure 3b. Under the excitation of 450 nm, the [(CH_3_)_4_N]_2−*x*_[(C_2_H_5_)_4_N]*_x_*MnCl_4_ phosphors all show green emission in the range of 450~610 nm. With the enhancement in n[(CH_3_)_4_N]/n[(C_2_H_5_)_4_N], the emission intensity of [(CH_3_)_4_N]_2−*x*_[(C_2_H_5_)_4_N]*_x_*MnCl_4_ phosphors gains a significant enhancement. When the n[(CH_3_)_4_N]/n[(C_2_H_5_)_4_N] ratio reaches 1, the as-prepared [(CH_3_)_4_N][(C_2_H_5_)_4_N]MnCl_4_ phosphors have the highest emission intensity. As the (CH_3_)_4_N/(C_2_H_5_)_4_N molar ratio further increases to 2, the emission intensity of [(C_2_H_5_)_4_N]_2_MnCl_4_ phosphors gradually declines.

To reveal the underlying reason for modulating the luminescence intensity, the distance adjustable Mn–Mn is calculated using the crystal structure of [(CH_3_)_4_N]_2_MnCl_4_, [(CH_3_)_4_N][(C_2_H_5_)_4_N]MnCl_4_ and [(C_2_H_5_)_4_N]_2_MnCl_4_ in Figure 1. The Mn–Mn shortest distance of [(CH_3_)_4_N]_2_MnCl_4_, [(CH_3_)_4_N][(C_2_H_5_)_4_N]MnCl_4_, and [(C_2_H_5_)_4_N]_2_MnCl_4_ is 7.89 Å, 9.02 Å, and 9.07 Å, respectively. The Mn–Mn shortest distance gradually augments with the increase in (C_2_H_5_)_4_N^+^ amounts, hindering the transfer of excitons between adjacent [MnCl_4_]^2−^ tetrahedra and boosting the exciton radiation recombination [29,31]. The PLQY value of the as-prepared phosphors increases from 13.48% of [(CH_3_)_4_N]_2_MnCl_4_ to 83.78% of [(CH_3_)_4_N][(C_2_H_5_)_4_N]MnCl_4_ (Figure 3d), which is higher than that of Mn^2+^-based metal halide phosphors reported in previous literature [28,29,31]. Unfortunately, the larger Mn–Mn distance leads to new nonradiative recombination. The (C_2_H_5_)_4_N^+^ ligands have longer carbon chains and a greater number of C−H bonds than (CH_3_)_4_N^+^ ligands, giving [(C_2_H_5_)_4_N]_2_MnCl_4_ higher vibrations, and resulting in a lower emission intensity [40]. The PLQY value of the as-prepared phosphors decreases to 44.01% for [(C_2_H_5_)_4_N]_2_MnCl_4_. As *x* increases, the FWHM of the as-prepared phosphors initially declines and then increases, and the emission peak position exhibits a blue shift from 520 nm to 516 nm, then a red shift to 518 nm (Figure 3c).

The PL decay curves of [(CH_3_)_4_N]_2−*x*_[(C_2_H_5_)_4_N]*_x_*MnCl_4_ phosphors are shown in Figure 3e. The PL lifetime was analyzed using a single exponential function [23]:

I(t) = I_0_ + A exp(−t/τ) (1)

where I(t) and I_0_ denote the emission intensity at time t and 0, respectively. A and τ are the constant and decay time, respectively. According to Equation (1), the lifetime values of [(CH_3_)_4_N]_2_MnCl_4_, [(CH_3_)_4_N][(C_2_H_5_)_4_N]MnCl_4_, and [(C_2_H_5_)_4_N]_2_MnCl_4_ are 0.733 ms, 3.786 ms, and 2.312 ms, respectively. The relatively long lifetime of [(CH_3_)_4_N]_2−*x*_[(C_2_H_5_)_4_N]*_x_*MnCl_4_ phosphors is attributed to the ^4^T_1_ → ^6^A_1_ transition of Mn^2+^ by the spin selection rule and the parity selection rule. This is similar to rare-earth ion-doped phosphors for application in displays and lightings [21]. The digital photographs of [(CH_3_)_4_N]_2−*x*_[(C_2_H_5_)_4_N]_x_MnCl_4_ phosphors with a different n[(CH_3_)_4_N]/n[(C_2_H_5_)_4_N] are shown in Figure 3f. Under daylight, the [(CH_3_)_4_N]_2−*x*_[(C_2_H_5_)_4_N]_x_MnCl_4_ phosphors exhibit a pale green color. In addition, the brightest green luminescence of [(CH_3_)_4_N][(C_2_H_5_)_4_N]MnCl_4_ phosphors can be visually observed under UV lamp irradiation, which is consistent with the results of the PL spectra and PLQY.

### 3.3. Emission Mechanism and Stability of [(CH_3_)_4_N][(C_2_H_5_)_4_N]MnCl_4_ Phosphors

**Figure 4:**
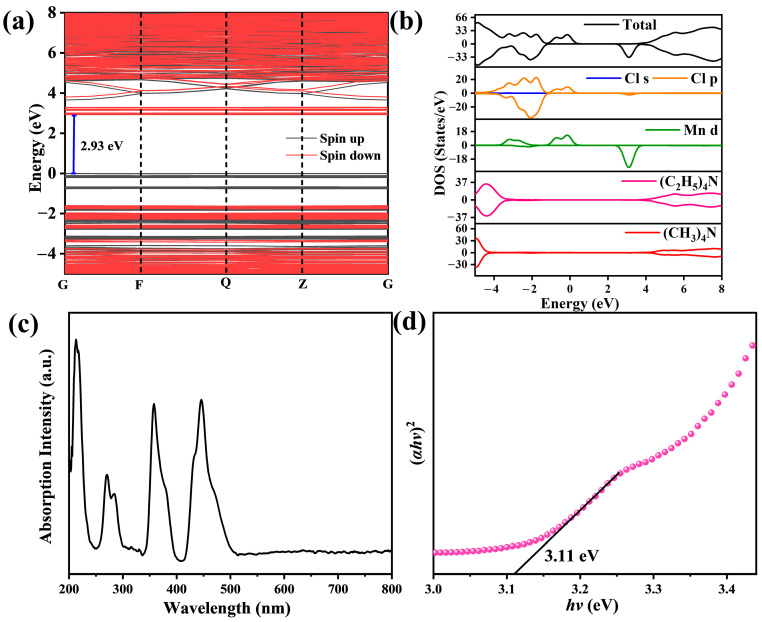
Band structure (**a**), total and partial density of states (**b**), absorption spectrum (**c**), and the optical band gap energy (**d**) of [(CH_3_)_4_N][(C_2_H_5_)_4_N]MnCl_4_.

To elucidate the emission mechanism of [(CH_3_)_4_N][(C_2_H_5_)_4_N]MnCl_4_, DFT was utilized to study the band and electronic structures. The band structure of [(CH_3_)_4_N][(C_2_H_5_)_4_N]MnCl_4_ is shown in Figure 4a. In order to provide a more accurate description of the interaction between Mn 3d electrons, the calculations took into account the spin-polarized magnetism. The calculated band gap of [(CH_3_)_4_N][(C_2_H_5_)_4_N]MnCl_4_ is 2.93 eV. The valence band maximum (VBM) is mainly contributed by the Mn d and Cl p orbitals, while the conduction band minimum (CBM) is mainly composed of Mn d orbitals (Figure 4b). It can be found that Cl p orbitals are mixed into the Mn d orbitals by Mn-Cl bonds, which is beneficial for relaxing the forbidden d electrons’ transition [20]. The partial density of states (PDOS) of (CH_3_)_4_N^+^ and (C_2_H_5_)_4_N^+^ are located at a relatively large energy gap. These results indicate that there is no obvious coupling between organic cations and [MnCl_4_]^2−^. In other words, the green emission of [(CH_3_)_4_N][(C_2_H_5_)_4_N]MnCl_4_ comes from the d–d orbital transitions of Mn^2+^. Moreover, the band fluctuation of VBM and CBM in the Brillouin zone is quite gentle, indicating a highly localized nature of the Mn 3d electrons. This is conducive to inhibiting the migration of excitation energy in adjacent luminescence centers of Mn^2+^, which is beneficial for achieving efficient green emission [20].

**Figure 5:**
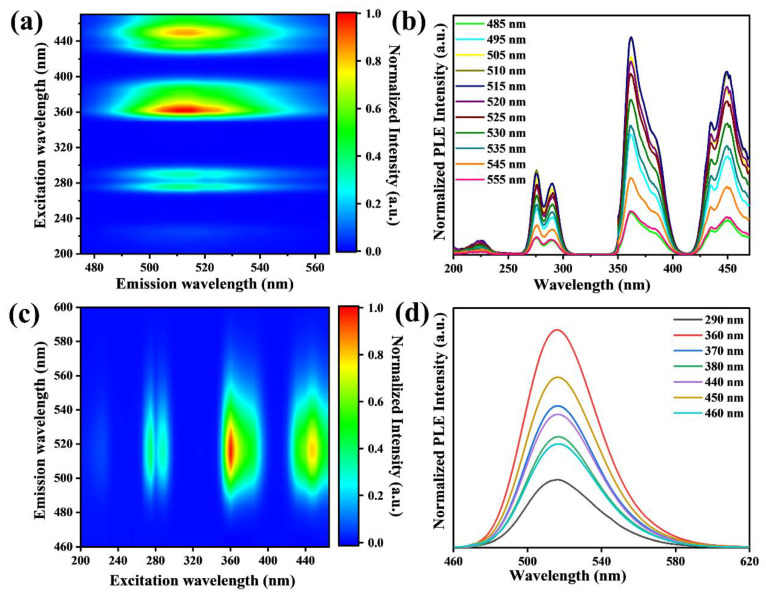
Pseudo-color contour map of PLE intensity vs. emission and excitation wavelengths of [(CH_3_)_4_N][(C_2_H_5_)_4_N]MnCl_4_ phosphors (**a**), PLE spectra of [(CH_3_)_4_N][(C_2_H_5_)_4_N]MnCl_4_ phosphors with different emission wavelength (**b**), pseudo-color contour map of PL intensity vs. emission and excitation wavelengths of [(CH_3_)_4_N][(C_2_H_5_)_4_N]MnCl_4_ phosphors (**c**), and PL spectra of [(CH_3_)_4_N][(C_2_H_5_)_4_N]MnCl_4_ phosphors with different emission wavelength (**d**).

The absorption spectrum of [(CH_3_)_4_N][(C_2_H_5_)_4_N]MnCl_4_ phosphors is displayed in Figure 4c. Four absorption bands of [(CH_3_)_4_N][(C_2_H_5_)_4_N]MnCl_4_ phosphors can be observed. The direct band gap value calculated by the Tauc relation is 3.11 eV, which is roughly in agreement with the PLE spectra of [(CH_3_)_4_N][(C_2_H_5_)_4_N]MnCl_4_ phosphors (Figure 4d). The optical bandgap is larger than the DFT-calculated bandgap (Figure 4a), due to the underestimation of band gaps from the PBE functional calculations. The pseudo-color contour map of the emission wavelength dependent on the PLE intensity and excitation wavelength is shown in Figure 5a and the PLE spectra of [(CH_3_)_4_N][(C_2_H_5_)_4_N]MnCl_4_ with different emission wavelengths are depicted in Figure 5b. The spectra of [(CH_3_)_4_N][(C_2_H_5_)_4_N]MnCl_4_ phosphors are nearly the same with different emission wavelengths, and there is no obvious shift in the PLE peak position. Additionally, as shown in Figure 5c,d, the emission spectra of [(CH_3_)_4_N][(C_2_H_5_)_4_N]MnCl_4_ phosphors display almost the same outline with varying excitation wavelengths. The emission peak position of [(CH_3_)_4_N][(C_2_H_5_)_4_N]MnCl_4_ phosphors also remains unchanged. These results further demonstrate that the green emission of [(CH_3_)_4_N][(C_2_H_5_)_4_N]MnCl_4_ phosphors is from intrinsic features rather than defect state luminescence.

**Figure 6:**
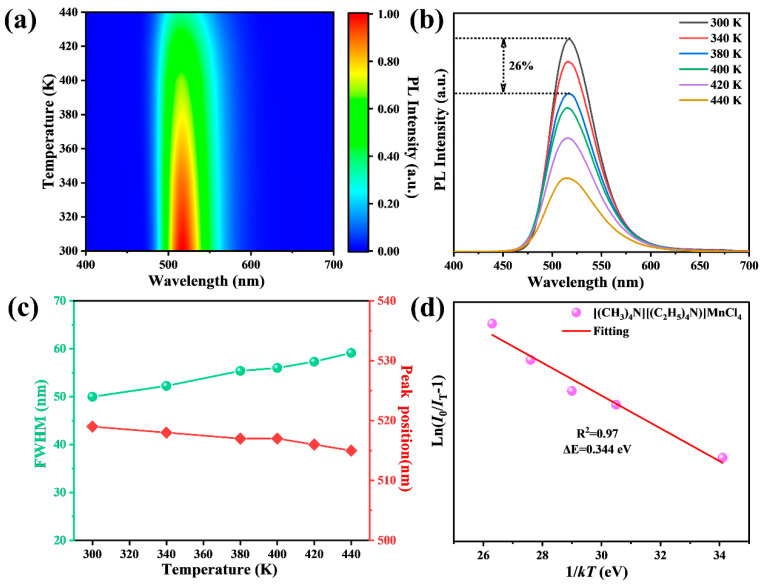
Pseudo-color contour map of PL intensity vs. temperature and wavelengths (**a**), temperature-dependent PL spectra of [(CH_3_)_4_N][(C_2_H_5_)_4_N]MnCl_4_ (**b**), the evolution of FWHM and peak position with temperature (**c**), and the thermal activation energy of [(CH_3_)_4_N][(C_2_H_5_)_4_N]MnCl_4_ calculated by using the Arrhenius equation (**d**).

Thermal stability is crucial for the practical application of metal halide materials. The pseudo-color contour map and temperature-dependent PL spectra of [(CH_3_)_4_N][(C_2_H_5_)_4_N]MnCl_4_ phosphors are illustrated in Figure 6a and 6b, respectively. With the elevation of temperature from 300 K to 440 K, the emission intensity of [(CH_3_)_4_N][(C_2_H_5_)_4_N]MnCl_4_ phosphors gradually decreases due to the increased interaction between the exciton and phonon [41]. Furthermore, the emission intensity of [(CH_3_)_4_N][(C_2_H_5_)_4_N]MnCl_4_ phosphors at 380 K still maintains 74.0% of that at 300 K, showing good thermal stability compared with the guanidine-based and ammonium-based metal halides previously reported [42,43,44]. As the temperature rises, the FWHM of [(CH_3_)_4_N][(C_2_H_5_)_4_N]MnCl_4_ phosphors gradually broadens (Figure 6c), which is attributed to an increase in the electron–phonon coupling effect. Additionally, the PL emission peak position of [(CH_3_)_4_N][(C_2_H_5_)_4_N]MnCl_4_ phosphors exhibits a blue shift as the temperature increases. This is because the lattice expansion increases the distance between adjacent Mn^2+^ ions, leading to a reduction in the spin−spin coupling energy and crystal field strength [41].

To reveal the thermal stability, the activation energy (ΔE) of [(CH_3_)_4_N][(C_2_H_5_)_4_N]MnCl_4_ phosphors is fitted by the Arrhenius formula [45]:

I_T_/I_0_ = [1 + Aexp(ΔE/kT)]^−1^ (2)

where I_T_ and I_0_ represent the intensity at temperature T and initial temperature, respectively, k represents the Boltzmann constant (k = 8.617 × 10^−5^ eV·K^−1^), and A represents a constant. According to Equation (2), the ΔE of [(CH_3_)_4_N][(C_2_H_5_)_4_N]MnCl_4_ phosphors is 0.344 eV (Figure 6d). The relatively large activation energy indicates the excellent thermal stability of [(CH_3_)_4_N][(C_2_H_5_)_4_N]MnCl_4_ phosphors [45].

### 3.4. Application in WLED for Wide Color Gamut Display

**Figure 7:**
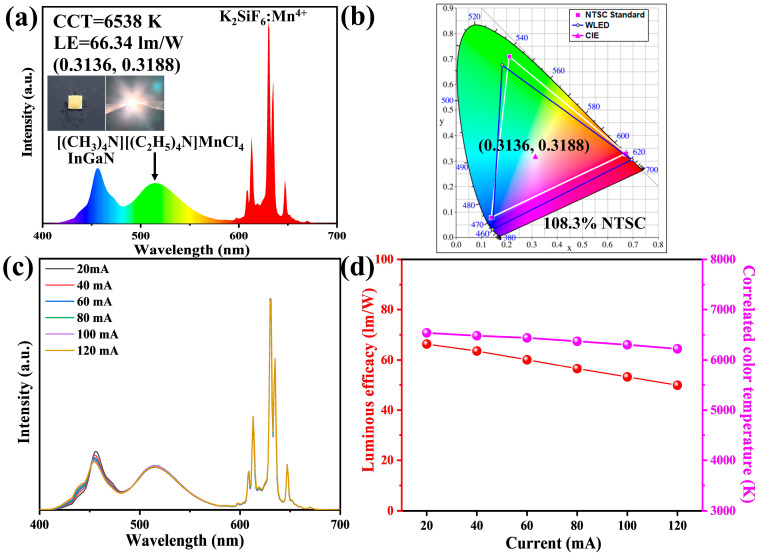
Emission spectrum of the fabricated WLED device based on [(CH_3_)_4_N][(C_2_H_5_)_4_N]MnCl_4_ green phosphor at 20 mA and the inset-showed photographs (**a**), the color gamut and chromaticity coordinate in the CIE 1931 system (**b**), emission spectra of the WLED with different driving currents (**c**), and the evolution of luminous efficiency and correlated color temperature with different driving currents (**d**).

The efficient green emission of [(CH_3_)_4_N][(C_2_H_5_)_4_N]MnCl_4_ phosphors under blue light excitation shows great potential for use in solid-state display and lighting applications. A WLED was fabricated using the [(CH_3_)_4_N][(C_2_H_5_)_4_N]MnCl_4_ green phosphors, combined with commercial K_2_SiF_6_:Mn^4+^ red phosphors and a blue LED chip. Figure 7a shows the emission spectrum and photographs of the WLED at a current of 20 mA. The fabricated LED device emits white light with a correlated color temperature (CCT) of 6538 K and a chromaticity coordinate of (0.3136, 0.3188), which is close to the standard white CIE coordinate of (0.33, 0.33). The WLED device has a high luminous efficiency of 66.34 lm/W. The color gamut of the WLED device based on the CIE 1931 color space is calculated to be 108.3% of the NTSC standard (Figure 7b), indicating that the as-fabricated WLED based on [(CH_3_)_4_N][(C_2_H_5_)_4_N]MnCl_4_ green phosphors has a wide color gamut. Importantly, the stability of the WLED with a driving current ranging from 20 to 120 mA was studied, as shown in Figure 7c,d. The green emission intensity of the WLED device gradually enhances and then slightly declines with the increase in the driving current. The green emission intensity of the WLED device at a driving current of 100 mA can maintain its initial intensity of 100%. The luminous efficiency and CCT of the WLED device only drop by 25.2% and 4.8%, respectively, demonstrating good operation stability. The wide color gamut and good stability of the as-fabricated WLED device based on [(CH_3_)_4_N][(C_2_H_5_)_4_N]MnCl_4_ green phosphors make it a promising option for use in solid-state lighting and display backlights.

## 4. Conclusions

In summary, the [(CH_3_)_4_N]_2−*x*_[(C_2_H_5_)_4_N]*_x_*MnCl_4_ phosphors were successfully synthesized via a facile mechanochemical method. Mixed-ligand engineering was employed to improve the emission properties of the phosphors. The effect of n[(CH_3_)_4_N]/n[(C_2_H_5_)_4_N] on the structure and PL properties was systematically investigated. The results indicate that the mixed-ligand strategy can significantly boost the emission intensity of the samples. The PLQY also improves from 13.48% of [(CH_3_)_4_N]_2_MnCl_4_ phosphors to 83.78% of [(CH_3_)_4_N][(C_2_H_5_)_4_N]MnCl_4_ phosphors. This is because mixed-ligand engineering modulates the adjacent [MnCl_4_]^2−^ distance, resulting in the enhanced d–d radiation recombination in isolated [MnCl_4_]^2−^ tetrahedra. The obtained [(CH_3_)_4_N][(C_2_H_5_)_4_N]MnCl_4_ phosphors exhibit a strong green emission at 516 nm with an FWHM of 45.53 nm. The stability results reveal that the [(CH_3_)_4_N][(C_2_H_5_)_4_N]MnCl_4_ phosphors show excellent thermal stability, maintaining 74.0% of the initial emission intensity at 380 K. Finally, the as-fabricated WLED device has a high luminous efficacy of 66.34 lm/W and a wide color gamut of 108.3% NTSC, indicating that [(CH_3_)_4_N][(C_2_H_5_)_4_N]MnCl_4_ green phosphors possess desirable application prospects in solid-state display backlights and lighting. This work provides new insight into modulating mixed A-site ligands to enhance PL properties, and offers inspiration for developing wide color gamut backlight display devices.

## Data Availability

The original contributions presented in the study are included in the article, further inquiries can be directed to the corresponding author.
